# Interdisciplinary evidence-based tumor board simulation training in surgical medical education

**DOI:** 10.1007/s00423-025-03856-9

**Published:** 2025-09-05

**Authors:** Nora Corinna Altmayer, Elias Khajeh, Johanna Fellhofer-Hofer, Anna Lintner, Matthias Alexander Fink, Constantin Schwab, Niels Halama, Felix Nickel, Arianeb Mehrabi, Fee Klupp

**Affiliations:** 1 Department of General, Visceral and Transplantation Surgery, University Hospital Heidelberg, Im Neuenheimer Feld 420, 69120 Heidelberg, Germany; 2 Department of General, Visceral, Vascular and Pediatric Surgery, Saarland University Hospital, Kirrberger Str. 100, 66421 Homburg, Germany; 3 Department of General, Visceral and Vascular Surgery, University Hospital, Wiener Neustadt, Corvinusring 3-5, Wiener Neustadt, 2700 Austria; 4 Clinic for Diagnostic and Interventional Radiology, University Hospital Heidelberg, Im Neuenheimer Feld 420, 69120 Heidelberg, Germany; 5 Institute of Pathology, University Hospital Heidelberg, Im Neuenheimer Feld 224, 69120 Heidelberg, Germany; 6 Medical Oncology, National Center for Tumor Diseases (NCT), Institute of Immunology, University Hospital Heidelberg, Im Neuenheimer Feld 460, 69120 Heidelberg, Germany; 7 Department of Translational Immunotherapy, German Cancer Research Center (DKFZ), Im Neuenheimer Feld 280, 69120 Heidelberg, Germany; 8 Department of General, Visceral and Thoracic Surgery, University Medical Center Hamburg – Eppendorf, Martinistr. 52, 20246 Hamburg, Germany

**Keywords:** Interdisciplinary training, Communication, Medical education, Tumor bord simulation, Evidence-based medicine

## Abstract

**Purpose:**

Evidence-based medicine (EBM), precision medicine and interdisciplinarity are becoming increasingly important in medical education. The purposeful connection between EBM and interdisciplinary work is given in the context of a tumor board, since therapy decisions are discussed here in an interdisciplinary team based on current evidence-based guidelines. To date, there has been little focus on either topic. Therefore, we aimed to assess the existing knowledge and offered a practical exercise in terms of a tumor board simulation training for medical students during their surgical study semester.

**Methods:**

First, a knowledge test was performed in 4th year medical students (*n* = 149) addressing the topic of EBM following an evaluation questionnaire. Subsequently, a structured one hour- lecture was held. The students were divided into groups of up to ten and assigned with specific professional roles of tumor board members. Thereafter, students participated in a live simulated tumor board. At the end the same knowledge test and evaluation questionnaire were delivered (*n* = 163). Study was conducted at Heidelberg Medical University between 2023 and 2024.

**Results:**

A significant increase in knowledge was seen before versus after the course only in the primarily better students (*p* < 0.0001). Almost no difference in knowledge test was seen before and after the course pertaining all students (*p* = 0.205). Evaluation questionnaire displayed that 45% (*n* = 66) of the students had not dealt with the topic of EBM before the course. 98% (*n* = 146) of the students considered that interdisciplinary work in medical profession is very important.

**Conclusion:**

Including EBM and precision medicine in a practical way into the medical curriculum is necessarily needed. As only the best students seemed to benefit significantly, a more sustainable approach might be the implementation of a longitudinal precision medicine curriculum.

## Introduction

Evidence-based medicine (EBM) is the conscious, explicit and prudent use of the current best evidence when making decisions about the care of individual patients – this was already stated in the 90´s [1]. Whereas precision medicine is called “the next generation of evidence-based medicine” focusing on individualized patient-centered therapeutic approaches [2, 3]. Multidisciplinary tumor boards (MTB) are an evidence-based approach to apply shared decision-making and patient safety concepts to medical practice. In daily clinical practice multidisciplinary tumor board-delivered treatment based on EBM is standard for cancer care. Multidisciplinary tumor boards are an evidence-based approach to apply shared decision-making and patient safety concepts to medical practice [4]. Moreover, Speccia et al. stated that multidisciplinary approaches are essential to ensure the complex care of cancer patients [5]. Interestingly, a positive influence of a higher number of interdisciplinary tumor boards on the clinical outcome has been proven [6]. Furthermore, interdisciplinary tumor boards have been associated with reduced mortality; e.g. Basendowah et al. demonstrated an overall mortality rate of 13% after two years in the MTB group versus 38% in the non-MTB group in gastrointestinal cancer patients (*p* = 0.08) [7]. Lamb et al. conducted a prospective study that evaluated MTB decision making with different MTB improvement interventions as checklists and team training. They could prove an amelioration in decision making [8]. Improving communication and team training of cancer teams also represents a current topic in the literature [9, 10]. Problem-based learning can also be found more and more in literature, as a scoping review showed that 49 out of 124 studies demonstrated better learning and knowledge acquisition through problem-based learning [11]. Regarding student’s education, inclusion of combined EBM, precision medicine and interdisciplinarity is quite not common and literature regarding a tumor board simulation training during medical education is scarce. Mattes et al. showed that students have deficits in oncology with a lack of understanding management basics to survivorship, radiation oncology and hospice and palliative medicine. But only an online survey was conducted in 76 students without any lecture beforehand [12]. Hasabo et al. published a study including 761 medical students showing a significant higher rating of all the items for EBM skills analyzed for example critical appraisal of available scientific literature, locating professional literature or identifying patient-relevant clinical questions. Students who received EBM training rated their skills better than those who didn’t receive it [13]. Van Woezik et al. demonstrated two attitudes in terms of “EBM task value” and self-efficacy in first-year undergraduate medical students during a 4-week EBM-course. The mean post-test scores for task value did not show a significant increase in either the control or the experimental group. However, both groups exhibited significantly higher scores in self-efficacy [14]. Another evidence-based medicine program received an EBM teaching over three years. The authors could show that different attitudes as ‘recalling prior knowledge’ or ‘practice of EBM’ were evaluated as sufficient by the students [15].

As several studies revealed that many students do not feel well prepared for the profession after their graduation implementation of EBM and precision medicine in a practical way could be a way for better preparation of students for residency [16, 17].Therefore, the purpose of our study was to integrate interdisciplinary activities as well as scientific competence like literature research combined with soft skills such as the precise presentation of complex patients and finding common compromises into the curriculum. We wanted to investigate whether a new established interdisciplinary course could be able to improve knowledge regarding EBM and precision medicine as well as to assess students’ attitudes towards interdisciplinary evidence-based skills in medical education.

## Materials and methods

### Study design

A prospective observational study design was used to evaluate the effectiveness of the educational intervention. The questionnaires were designed and developed by the authors, who are specialists in surgery, oncology or radiology.

### Study setting

The study was conducted at Heidelberg University between 2023 and 2024.

### Study population/sample size

Fourth-year medical students during their surgical semester were invited to participate voluntarily. A total of 149 students participated in the pre-test, and 163 students completed the post-test.

### Inclusion and exclusion criteria

Students currently enrolled in the medical curriculum and attending the tumor board simulation course were included. Those with incomplete data were excluded.

### Variables and outcomes

The primary outcome measured was the improvement in knowledge scores, while secondary outcomes included subjective evaluations on the Likert scale questionnaire.

### Survey

A validated Likert scale questionnaire assessed students’ confidence and perceived understanding of the topics covered.

The participants were not selected, participation was voluntary and anonymous. Tumor board simulation training began with a knowledge test with individual statements to be marked as true or false (*n* = 40 points, 90 s per question) on the topic of precision medicine and EBM and Likert scale evaluation questionnaire consisting of 10 questions. The questions of the knowledge test were prepared jointly by a specialist in visceral surgery, oncology, pathology and radiology. Directly after finishing the questionnaires, a specialist in visceral surgery gave an one-hour lecture on the topic of “EBM and precision medicine”. The lecture was theoretical, focusing for example on topics as definitions and types of studies. A preparation period at home with literature research for the latest evidence on real patient cases followed in groups of a maximum of 10 students. Students conduct the literature research at home in predetermined groups of a maximum of 10 students. Students were grouped randomly. Patient cases were examined by the same specialist in visceral surgery - who held the lecture before - together with a specialist in oncology. A few weeks later the practical session of the course took place. Each student took on the role of each physician represented in a tumor board (surgeon, oncologist, radiologist, radiotherapist, pathologist, nuclear medicine specialist) and the cases have been discussed in an interdisciplinary manner in 15-minute discussion rounds. The four patient cases addressed the topics of adenocarcinoma of the sigma, hepatocellular carcinoma, adenocarcinoma of the stomach and pancreatic neuroendocrine tumor with liver metastasis. Since there were four simulation groups at one day, the course lasted one hour. The simulation was carried out with the entire cohort. The other teams observed the performing group. Specialist physicians supervised the course. The same specialists held the lecture and supervised the tumor board teams. The course ended by performing the same knowledge query with an evaluation. All respondents were included in the study under the same conditions. The knowledge questions contained of K prim questions, this involves statements to be evaluated individually. For each statement, a decision had to be made as to whether it is true or false. The values of the individual answers were summed up and resulted in the final value of a maximum of 40 points. The evaluation questions were analyzed using a 5-point Likert scale (range from one = do not agree until five = totally agree with) [18]. To obtain a statement about the positive evaluations, the percentages of the numerical Likert scale of 1 and 2 were added together. To obtain a statement about the negative ratings, the percentages of the scale of 4 and 5 were added together accordingly. The Likert scale was used as self-assessment tool. We assessed personal attitudes regarding psychosocial skills.

The median was used to reflect the central tendency of responses, as Likert scale data are ordinal and not normally distributed. Study participation was voluntary, written informed consent was obtained from every participant and study was approved by local ethics committee (S-333/2023). Statistical analysis was performed using ExcelTM 2019, SPSSTM (version 29) and GraphPadPrismTM (version 9). Results of the knowledge test were reported as the 50% quartile range with 25% and 75% interquartile ranges, minimum and maximum. Mann-Whitney-U-Test was applied to calculate differences between independent samples. Mc Nemar`s test was applied to calculate differences between dependent dichotomized data. For evaluation questions Chi-square test and Fisher’s exact test was applied. Results were considered significant if p-value was < 0.05. Evaluation questions two and four could not be analyzed statistically because Likert range five was not selected by the students (Table 1).

**Table 1:** Contains a list of all evaluation questions

Evaluation question Q1	“Have you dealt with the topic of “evidence-based medicine” before the lecture?”
Evaluation question Q2	“I think it makes sense for several medical departments to deal with a particular patient at the same time.”
Evaluation question Q3	“I can read up on a patient`s oncological therapy and don`t need to consult with other departments.”
Evaluation question Q4	“How important is interdisciplinary work for you in the medical profession?”
Evaluation question Q5	“The tumor board is concerned with making a decision that is technically feasible and oncologically meaningful.”
Evaluation question Q6	“Finding a compromise is…”
Evaluation question Q7	“Speaking freely, discussing and arguing should be practiced more during studies.”
Evaluation question Q8	“Not being right is for me…”
Evaluation question Q9	“The more harmonious the team, the better the decisions.”
Evaluation question Q10	“Do you think you will benefit from the lecture and the practical exercise of a tumor board?”

## Results

### Knowledge exam

**Fig. 1:**
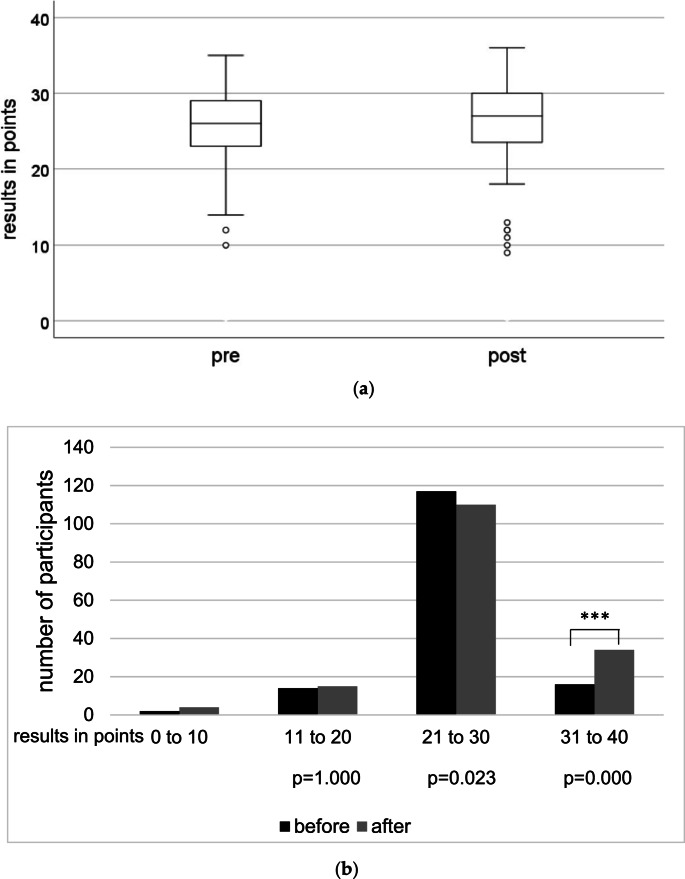
**a**) Test results in points before and after the seminar (*p* =0.205). The total result was 40 points. Bar represents median + minimum/maximum (n_before_= 149 and n_after_=163); (**b**) Number of participants according to results with 4 groups (1: 0-10 points, 2: 11-20 points, 3: 21-30 points, 4: 31-40 points)

A median of 26 out of 40 points were reached before the course, respectively 27 points afterwards (*p* = 0.205, Fig. 1.a).

A division according to results into 4 groups took place with group 1: 0–10 points (*n* = 2 pre, *n* = 4 post), group 2: 11–20 points (*n* = 14 pre, *n* = 15 post), group 3: 21–30 points (*n* = 117 pre, *n* = 110 post) and group 4: 31–40 points (*n* = 16 pre, *n* = 34 post) (Fig. 1.b). Significant difference was assessed between the initial and final scores of the successful students in group 4 (*p* < 0.0001).

As 60% of total results (complying 24 points) is often the pass mark of exams in medical studies, 71% of the students achieved equal or greater than 60% of total points before the course, respectively 74% after the course. ~30% of the students would not have passed the exam if it had been mandatory.

25th percentile represents 23 points for both groups, 75th percentile represents 29 points for the students who participated in the pre-test, and 30 points for the students in the post-test.

## Evaluation questionnaire

**Fig. 2:**
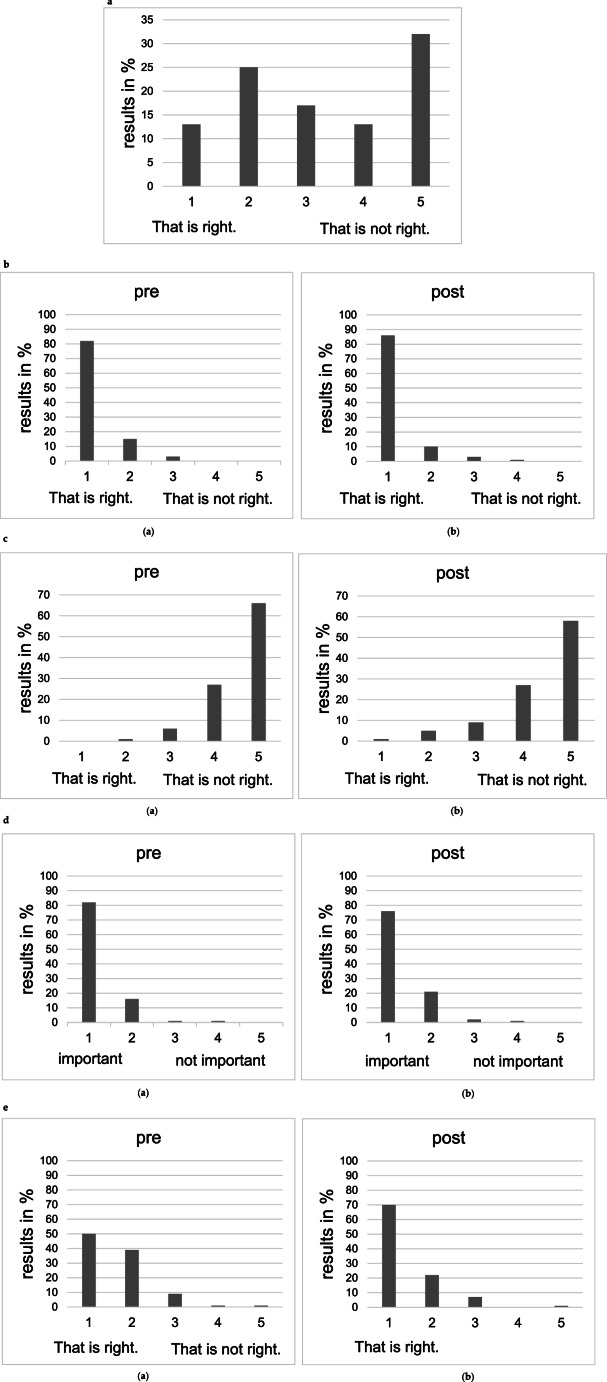

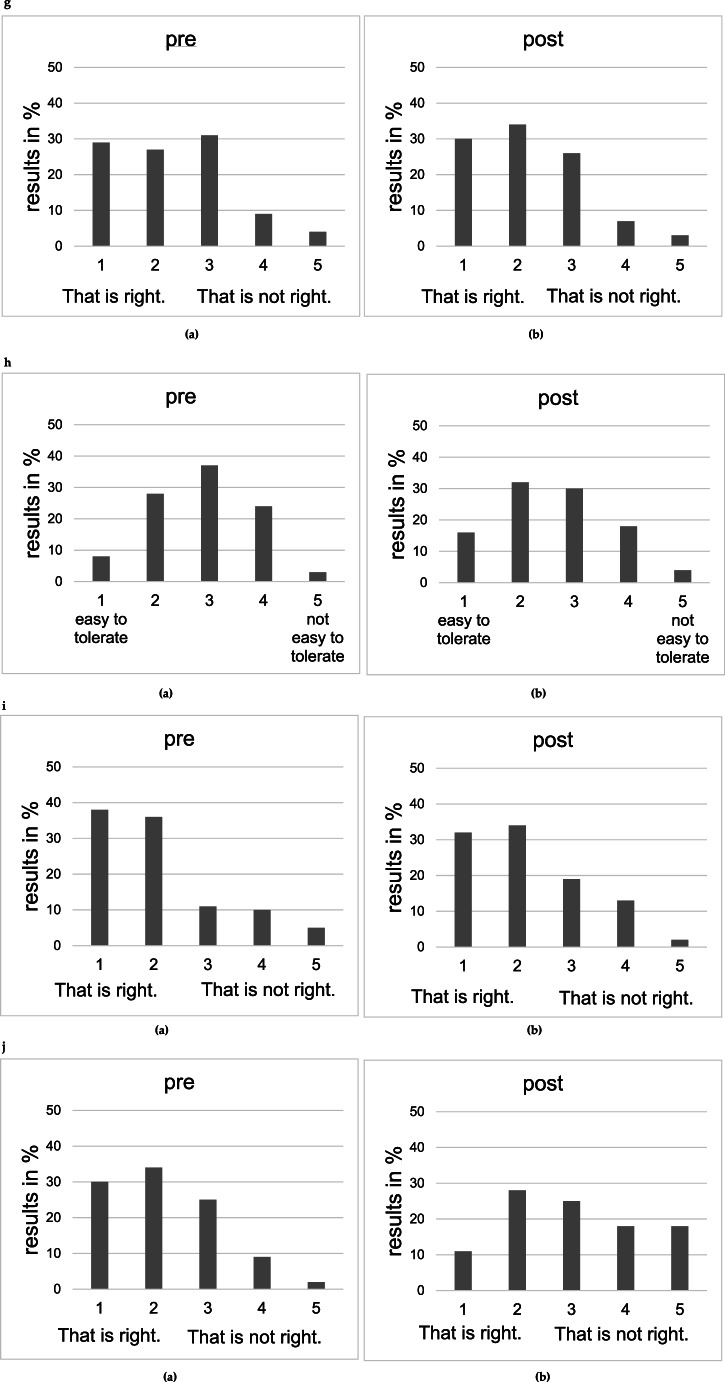
(**a**) Evaluation question Q1: “Have you dealt with the topic of “evidence-based medicine” before the lecture?”, evaluation before the lecture (n_total_= 149). **b** Evaluation question Q2: “I think it makes sense for several medical departments to deal with a particular patient at the same time.” (p = NA), evaluation before the lecture (**a**) (n_total_= 149) and evaluation after the tumor board simulation course (**b**) (n_total_=164). **c** Evaluation question Q3: “I can read up on a patient`s oncological therapy and don`t need to consult with other departments.” (*p* = 0.310), evaluation before the lecture (**a**) (n_total_= 149) and evaluation after the tumor board simulation course (**b**) (n_total_=164). **d** Evaluation question Q4: “How important is interdisciplinary work for you in the medical profession?” (p = NA), evaluation before the lecture (**a**) (n_total_= 148) and evaluation after the tumor board simulation course (**b**) (n_total_=164). **e** Evaluation question Q5: „The tumor board is concerned with making a decision that is technically feasible and oncologically meaningful.” (*p* = 0.053), evaluation before the lecture (**a**) (n_total_= 148) and evaluation after the tumor board simulation course (**b**) (n_total_=164). **f** Evaluation question Q6: „ Finding a compromise is…” (*p* = 0.002), evaluation before the lecture (**a**) (n_total_= 149) and evaluation after the tumor board simulation course (**b**) (n_total_=164). **g** Evaluation question Q7: “Speaking freely, discussing and arguing should be practiced more during studies.” (*p* = 0.799), evaluation before the lecture (**a**) (n_total_= 149) and evaluation after the tumor board simulation course (**b**) (n_total_=164). **h** Evaluation question Q8: “Not being right is for me…” (*p* = 0.323), evaluation before the lecture (**a**) (n_total_= 148) and evaluation after the tumor board simulation course (**b**) (n_total_= 162). **i** Evaluation question Q9: “The more harmonious the team, the better the decisions.” (*p* = 0.356), evaluation before the lecture (**a**) (n_total_= 149) and evaluation after the tumor board simulation course (**b**) (n_total_= 164). **j** Evaluation question Q10: “Do you think you will benefit from the lecture and the practical exercise of a tumor board?” (*p* = 0.001), evaluation before the lecture (**a**) (n_total_= 148) and evaluation after the tumor board simulation course (**b**) (n_total_=163)

On a 5-point Likert scale, 45% of the students stated that they had not dealt with the topic of EBM before the course (Question 1 = Q1, Fig. 2.a). 97% of the students before the course found it useful that different departments deal with a certain patient at the same time, after the course, again 96% quoted the same (Question 2 = Q2, Fig. 2.b, not analyzable). Moreover, the statement “I can read up on a patient’s oncological therapy and don’t need to consult with other departments” was negatively confirmed by 93% of the students before the course and by 85% after the course (Question 3 = Q3, Fig. 2.c, *p* = 0.310). Furthermore, 98% of the students stated that interdisciplinary work in medical profession is very important to them. After the course, 97% of the students confirmed this again (Question 4 = Q4, Fig. 2.d, not analyzable). The fact that tumor board is about a decision that is technically feasible and oncologically meaningful was affirmed by 89% of the students before and by 92% of the students afterwards (Question 5 = Q5, Fig. 2.e, *p* = 0.053). Finding a compromise was rated less negatively by the students after the course than before. 53% found it difficult to find compromises before, while 34% found it difficult to find compromises afterwards (Question 6 = Q6, Fig. 2.f, *p* = 0.002). Even before the course, 56% of the students stated that speaking, discussing and arguing freely should be practiced more during their studies. After the course, 64% of the students shared this opinion (Question 7 = Q7, Fig. 2.g, *p* = 0.799). Besides, the course helped the students dealing with not being right. Before the course, 36% of the students quoted that being wrong was easy to bear, after the course it was 48% (Question 8 = Q8, Fig. 2.h, *p* = 0.323). The statement “The more harmonious the team, the better the decisions.” was positively confirmed by 74% of the students before and by 66% of the students after the course (Question 9 = Q9, Fig. 2.i, *p* = 0.356). Finally, the question “Do you think you will benefit from the lecture and the practical exercise of a tumor board?” was answered positively by 64% of the students before the course and by 39% of the students after the course (Question 10 = Q10, Fig. 2.j, *p* = 0.001).

The groups differed in the number of students because more students attended the tumor board simulation than the lecture. Moreover, participation was voluntary and anonymous; therefore matching of pre and post results of each student was not possible, too.

A “team leader” has not been selected.

Summarized, in the knowledge test only the primarily better students benefited. The most important results arising from the evaluation questionnaire were the statement that nearly 100% of the students before after the seminar indicated that interdisciplinary work in the medical profession is very important to them. The course appeared to encourage psychosocial skills, as finding compromises was easier for the students after the seminar and being wrong was better to accept after the course.

## Discussion

In our study, we could demonstrate that, although the whole study population failed in achieving significantly better results, the successful students achieved a significant better final result in the knowledge test. About 30% of the students would have failed the course if it had been a real exam and we could show that 45% of the students had not dealt with the topic of precision medicine before the course. This course offers the opportunity to train several soft skills such as analytical thinking, empathy, teamwork, critical thinking, and commitment. This goes in line with our study results as 53% of the students found it difficult to find compromises before, while only 34% found it difficult to find compromises afterwards and 36% of the students quoted before that being wrong was easy to bear, respectively 48% afterwards. Critical thinking skills were also positively affected by the course. Another positive aspect of this course is the finding that interdisciplinarity in medicine is of great relevance to students, almost 100% of the students stated so before and after the course. However, one could argue that the lack of change in the students’ behaviour means a slight impact of the course on this aspect. Besides, the course also cleared that speaking freely, discussing and arguing is not yet practiced enough and the students became aware of this during the course. Tumor boards are an important component of curative and palliative cancer management with proven benefits in patient outcomes [4, 12, 19, 20]. However, till now the subject of tumor board simulation training has rarely been found in medical education. Regarding the literature, EBM and precision medicine studies are scarce. LaRieviere et al. introduced a multidisciplinary oncology curriculum were medical students participated in a series of lectures, an approach to breaking bad news, guideline-based management and multidisciplinary tumor board simulation. However, compared to our study it was conducted for final-year medical students, but also for students up to the first year which seems to be an early stage in medical training for these issues. Data of the number of participants are missing and it is only an opinion piece without any statistical results [21]. Another two articles dealt with young, graduated oncologists and medical students in general who practiced presenting challenging patient cases with experts in international or European courses to improve their oncology knowledge and clinical skills [22, 23]. Contrary to our study, students were preselected and were in their final two last years of medical studies [23]. Why a difference between the groups in terms of “EBM task value” could not be demonstrated in the above-mentioned Dutch study could be explained through an inadequate educational design [14]. Elçin et al. performed an evidence-based medicine program over three years. Finally, different attitudes were evaluated positively which raises the question whether our study should have lasted longer [15]. At the same time as our study was carried out, a similar study was conducted at the University Hospital of Munich. In comparison to our study, more lectures were offered as this was an one-week course. However, the students were not allowed to perform the tumor board simulation on their own under supervision. Five expert lecturers were presenting the tumor board cases and afterwards students decided on their recommendation for a possible treatment. The authors could show a significant improvement in comprehension of interdisciplinary clinical decision-making which underlines the necessity of these courses [24].

It is tempting to speculate why there was almost no improvement in the results of the knowledge test before and after the tumor board simulation training regarding the medium-grade and lower-grade students. First, it is a matter of material that had not yet been taught to them and that was not repeated. The lack of knowledge gain could be due to other reasons, for example the course content as it was a demanding seminar. Also, the unknown teaching methods, students’ prior knowledge and motivation could be factors for the missing improvement in the results of the knowledge test. Another reason for the rather unfavourable results before and after the test could be the use of K prim questions, as these are more difficult to answer than multiple choice questions. One reason might be that the better students belong to the group that has dealt with the topic before. Since the course content is complex, prior knowledge helped them to understand the content and perform better after the course.

Some limitations of our study have to be mentioned. First, the study was conducted at a single institution. Due to the lecturers’ limited experience to date, the quality of a new teaching method may not correspond to the quality of courses that have been in place for many years. Student motivation could also be a reason for the poor knowledge and evaluation test results. The students may have felt overwhelmed by the new learning content. An incompatible curriculum alignment could also play a role. A tumor board requires that the subjects involved are well known by the students. Possibly, this course should be offered one year later in the curriculum. Despite these reasons, a curriculum alignment with more interdisciplinary practical courses seems useful in order to better prepare students for professional life. Our study does not demonstrate the improvement of interdisciplinary evidence-based skills. An evaluation questionnaire was used to assess students’ attitudes toward different topics, but an objective assessment of skill improvement should be carried out in further studies.

Another limitation was the missing assessment of quality of teamwork. To score teamwork during MTB meetings, Lamp et al. used a specific assessment tool called the Metric of Decision Making with the aim to enhance the ability of a tumor board to reach treatment decisions. The authors utilized a standardized behavioral marker system with descriptive end points [8]. With a comparable tool, a more accurate evaluation of the team’s work could have taken place.

A further limitation is that due to the anonymous data acquisition no pre versus post matching of each student could be made. In principle, generalizability of this study is given because tumor boards, evidence-based and precision medicine are performed in every hospital. So independently from the study location it can be reproduced.

We postulate the effect that tumor board simulations ran more effectively when a team leader was established within the group of students. It is mentioned that a good leadership is a prerequisite for effective teamwork [10, 25, 26] and MTBs need a leader to encourage full participation of team members [27–29]. Ruhstaller et al. [27] described different key leadership skills needed to create productive discussions, like the ability to stop individuals from encourage their own self-interest. Also, leadership clarity represents an important role in the field of MTBs and it is assumed that there could be an association with clear team objectives and high levels of participation [25]. Therefore, a team leader should be appointed before the course.

Referring the evaluation question, if students think they would have benefited from the lecture and the practical exercise fewer students felt they had benefited from the course afterwards in comparison to the evaluation results before. The reason could be that some students may felt overwhelmed by the new teaching approach. Subsequently, offering future students the opportunity to visit a real tumor board before the course could minimize this problem. Therefore, the practical utility could be improved with a more sustainable approach in the sense of implementation of a longitudinal curriculum consisting of multiple sessions as a participation of a real tumor board, a lecture in the beginning, theoretical seminars with case studies and repetition of the lecture content, evidence-based literature research and practical seminars followed by a feedback round. Applying medical-scientific skills in a problem-related and independent manner is required from the students. However, practical implementation showed that some students were unacquainted in being participants in a discussion, others discussed confidently without having any fears.

In summary, data indicates a knowledge gap before the test and a deficit in acquired knowledge after the test. However, the students assessed some interdisciplinary and psychosocial aspects more positively than before the seminar.

## Conclusions

Since German medical studies require more interdisciplinary courses and EBM is a complex subject to learn, a tumor board offers a good opportunity to integrate both topics into medical studies in a practical way. This study demonstrates the implementation of a tumor board simulation training in the curriculum of medical education in fourth year students. Future studies should expand this program to increase learning success in a longitudinal way and to prepare students for the profession after their graduation regarding both scientific competences and soft skills. The integration of tumor board simulation training offers a promising method of integrating interdisciplinary activities into the curriculum.

## Data Availability

The data presented in this study are available on request from the corresponding author due to privacy.
